# Thiamine reduced metabolic syndrome symptoms in rats via down-regulation of hepatic nuclear factor-kβ and induction activity of glyoxalase-I

**DOI:** 10.22038/ijbms.2021.53707.12086

**Published:** 2021-03

**Authors:** Sina Mahdavifard, Razieh Dehghani, Farhad Jeddi, Nowruz Najafzadeh

**Affiliations:** 1 Department of Clinical Biochemistry, Ardabil University of Medical Sciences, Ardabil, Iran; 2 Department of Genetics and Pathology, School of Medicine, Ardabil University of Medical Sciences, Ardabil, Iran; 3 Research Laboratory for Embryology and Stem Cells, Department of Anatomical Sciences, School of Medicine, Ardabil University of Medical Sciences, Ardabil, Iran

**Keywords:** Glycation, Glyoxalase-I, Metabolic syndrome, Nuclear factor- kβ, Thiamine

## Abstract

**Objective(s):**

Metabolic syndrome (MS) is a cause of death worldwide. The hepatic nuclear factor- NF-kβ (NF-kβ) is the cardinal player of hepatic homeostasis, insulin sensitivity, and lipid metabolism. Thus, we investigated the effect of thiamine on hepatic gene expression of NF-kβ and its levels of activators in MS rats.

**Materials and Methods:**

Male Wistar rats were randomly divided into 4 equal groups (ten rats in each group): normal, MS, and two alike groups under thiamine treatment. MS was induced in rats with a high sucrose solution (40 % in drinking water) for 4 months. Treated groups of rats received 0.18 % of thiamine daily in drinking water. Hematoxylin-Eosin stains were employed to determine the histopathological changes of the liver. Metabolic profile, glycation products, oxidative stress, inflammatory markers, the activity of glyoxalase-I, as well as NF-kβ hepatic expression of all rat groups, were determined.

**Results:**

Acute hepatitis was not observed in the livers of the thiamine treated MS rats. Besides, the treatment showed an advantageous effect on glucose, lipid metabolism, and body weight via down-regulation of hepatic NF-kβ and induction of glyoxalase system activity. Furthermore, the treatment decreased diverse glycation, oxidative stress, and inflammatory markers (*P*>0.001).

**Conclusion:**

Thiamine decreased body weight and improved metabolism and activity of glyoxalase-I in MS rats with anti-glycation, antioxidant, and anti-inflammatory activities. Further, the treatment had a hepato-protective effect via reduction of NF-kβ signaling.

## Introduction

Metabolic syndrome (MS) or insulin resistance syndrome is a cause of death worldwide (1-3). MS is marked by insulin resistance, abdominal obesity, dyslipidemia, increased blood pressure, hypercholesterolemia, and a pro-inflammatory state (2). Obesity is one of the primary risk factors for MS and type-2 diabetes. Hepatic inflammation or metabolic inflammation participates in insulin resistance, MS, type-2 diabetes, cardiovascular disease, and neurocognitive dysfunction. The hepatic nuclear factor- NF-kβ (NF-kβ) is the cardinal player of hepatic homeostasis, stress responses (4), and insulin sensitivity. Free fatty acids (FFAs), early to end glycation products, cytokines, and oxidized LDL activate the NF-kβ pathway leading to insulin resistance, glycemia, and dyslipidemia (5, 6). Obesity and inflammation could be successive causative factors, and using anti-inflammation compounds is an effective strategy to oppose obesity and MS (7, 8). 

Early, intermediate, and end glycation products as sources of oxidative stress and inflammation contribute to reduction of insulin secretion, insulin resistance, and vascular complications (9-11). Methylglyoxal (MGO) as an intermediate glycation product is a potent intracellular glycating agent that forms advanced glycation endproducts (AGEs). The level of sera AGEs in overweight people is higher than normal and a risk factor for MS (12). The activity of the glyoxalase system is the principal defense against the accumulation of MGO ameliorating pathological conditions like insulin resistance (13, 14) and diabetes. Thus, recently reduction of glycation products and induction of activity of the glyoxalase system as an effective treatment strategy for vascular complications is considered (10, 15). 

Thiamine (vitamin B1) is essential for ATP, certain neurotransmitters, nucleic acids, lipids, amino acids, steroids, and glutathione synthesis. Further, it is vital for metabolism (glucose and lipid) and normal organ function. It has antioxidant, anti-glycation, and anti-inflammatory activities (10). Thiamine deficiency is common in MS (16). It causes hyperglycemia, insulin resistance, (10) hypertension (17), oxidative stress, and inflammation, as well as elevation of glycation products (10). Based on literature review, there is evidence only about the effect of thiamine in the rat model of obesity on body weight and metabolism disorders (18). Thus, in this study, we investigated for the first time the effect of thiamine on hepatic expression of nuclear factor-kβ (NF-kβ), and the activity of glyoxalase-I (Glo-I) in the MS rat model. Furthermore, the effect of treatment on glucose and lipid metabolism and glycation, oxidative stress, and inflammatory markers were studied.

## Materials and Methods

***Materials***

All materials were of analytical grade and purchased from Sigma or Merck Chemical Companies**.**

***Rat model of metabolic syndrome***

Male Wistar rats weighing 160±10 g, were purchased from the Faculty of Veterinary Medicine, Tehran University, Iran. Animals were kept under controlled conditions with free access to food and water. This study was approved by the Ethics Committee of Ardabil University of Medical Sciences (identification code ‘IR.ARUMS.REC.1397.272’). After two weeks, forty male Wistar rats were casually allotted into four groups of normal rats (N), MS rats, and two identical ones under thiamine treatment: N (Thiamine) and MS (Thiamine). MS was induced in rats with a solution of sucrose 40 % in drinking water for 4 months (19) and the treated groups received 0.18% of thiamine hydrochloride (THC) in drinking water daily for four months. The dose of the treatment was chosen according to our recent study (10) and the literature (20). All groups were fed a standard chow diet. 

 At the end of the experiment, the weight of all rats was measured. Then, the percentage of alteration of body weight of rats after 4 months compared with start of the study was calculated. After 16 hr of fasting, blood samples were collected from their hearts and transferred into test tubes with and without EDTA after anesthetizing with IP injection of ketamine & xylazine (respectively, 90 and 10 mg/kg body mass). Serum samples were prepared by 10 min centrifugation of blood at 1500g, 4 °C and stored at −70 °C until measurements. Their livers were removed and weighed instantly.

*Determination of biochemical parameters*

Fasting blood glucose (FBG), triglyceride (TG), total cholesterol (TC), LDL, HDL, alanine transaminase (ALT), and aspartate transaminase (AST) were measured by utilizing commercial kits (Pars Azmoon, Tehran, Iran). The cardiovascular indices were determined with the calculation of LDL/HDL, TC/HDL, TG/HDL, and TG/FBG ratios. Serum FFA levels were measured with high-pressure liquid chromatography (HPLC) within one hour after rehydration (21).

The serum insulin level was determined by the enzyme-linked immunosorbent assay (ELISA) method using a rat insulin kit (ZellBio GmBH, Germany). Moreover, HOMA1 or HOMA-β (homeostasis model assessment of insulin resistance) was determined using the equation below (22):

HOMA1=Fasting glucose mmol1×Fasting insulin (μUml)22.5

Also, HOMA2, beta-cell function (%B), and insulin sensitivity (%S) were determined with the HOMA2 calculator software released by the Diabetes Trials Unit, University of Oxford (23). 

*Determination of glycation products in serum and the activity of Glo-I in hemolysate*

Glycated albumin (g-Alb) was assayed with a colorimetric method based on the reduction of nitroblue tetrazolium chloride, and absorbance was read at a wavelength at 530 nm (24). Glycated LDL was measured based on the formation of hydroxymethyl furfuraldehyde chromogen (25). Methylglyoxal (MGO) was assayed by a reverse phase HPLC (26). AGEs measured in the serum of rats via the determination fluorescence intensity was recorded at the emission maximum (440 nm) upon excitation at 370 nm (27). The activity of glyoxalase-I (Glo-I) in hemolysate was detected by measuring the initial formation rate of S-D-lactoylglutathione and the activity of the enzymes expressed as unit/ml (U/ml) (28).

*Determination of oxidative stress and inflammatory markers in serum*

The oxidative stress markers as malondialdehyde (MDA) were measured based on determination of the absorbance of thiobarbituric acid at 532 nm. Advanced oxidation protein products (AOPP) were assayed with spectrophotometric detection, according to the method of Witko-Sarsat *et al*. (29). Early products of LDL oxidation were measured spectrometrically at 234 nm (30). Fluorescence intensity of end oxidation product of LDL was detected at the emission maximum of 430 nm upon excitation at 360 nm (31). Reduced glutathione (GSH) was analyzed with UV-HPLC at 210 nm (32). The activity of paraoxonase-I (PON1) was done with a spectrophotometer by measuring the p-nitrophenol absorbance within one minute at a wavelength of 412 nm. P-nitrophenol was obtained by paraoxon hydrolysis (33). The sera activity of CAT was measured with a modified Abi method (34). Briefly, 5 µl serum was added to a mixture (containing 10 mm of H2O2 in equal volume phosphate buffer 50 mm, pH= 7 and saline) and absorbance read at 240 nm until 20 sec.

Inflammatory markers as IL-1β were determined using the ELISA kit (ZellBio GmBH, Germany). The sera activity of myeloperoxidase (MPO) was measured by reading the absorbance of oxidized guaiacol at wavelength 470 nm. Briefly, 10 µl serum was added to a mixture (50 mM potassium phosphate buffer with 100 mM guaiacol and 0.0017% (w/w) hydrogen peroxide, pH 7.0 at 25 °C) and absorbance was read at 470 nm until 4 min. 

*Gene expression of hepatic NF-* *κB*

Total RNA was extracted from hepatic tissue using TRIzol reagent (Invitrogen, USA). The purity and concentration of extracted RNA were detected at 260 nm and purity was determined by 260/280 nm ratio with a NanoDrop spectrophotometer. cDNA was synthesized by reverse transcription following the manufacturer’s protocols (MBI Fermentas, Lithuania). qRT-PCR was performed with a standard SYBR-Green PCR kit (Toyobo, Japan), and gene-specific PCR amplification was performed using an ABI 7300 (Applied Biosystems, Germany). Β-actin (ACTB) was used as a housekeeping gene for normalization of gene expression data. RT- PCR primer sequences were as follows: 

NF-kβ: 5´-CCTGTCTGCACCTGTTCCAA-3´ (forward)

3´ACTCCTGGGTCTGTGTTGTT-5´(reverse) 

ACTB: 5´-GGAGAA GATTTGGCACCACACT-3´ (forward) 

3´-CGGTTGGCCTTAGGGTTCAGA-5´ (reverse). 

Relative gene expression levels were calculated using the 2^- ΔΔCT^ method after normalization to the mRNA level of β-actin. The first Δ*CT *is the difference in threshold cycle between the target and reference genes: ΔCT= CT (NF-kβ)-CT (ACTB) (35).

*Pathological study*

Sections of liver samples were fixed in a buffer solution containing 10% formalin and processed for paraffin embedding. Then, sections were stained with Hematoxylin-Eosin (H&E) and observed using light microscopy for histopathological parameters (36).

***Statistical analysis***

All data were expressed as mean±SD (standard deviations). The Kolmogorov-Smirnov test represented the normal distribution of the results which was the reason for using parametric methods. Multiple analysis of variance (MANOVA-TUKEY) test was used to compare different variables used in all four groups using SPSS (ver. 16). Statistical, significance was defined as *P<*0*.*05.

## Results

The comparison of the percentage of body weight alteration after four months and glucose and lipid metabolism in treated and untreated normal and MS rats are represented in Table 1. Induction of MS in rats via sucrose solution consumption for four months increased body weight, FBS, HOMA1, and HOMA2, as well as decreasing β-cell function (%B) and insulin sensitivity (%S). Furthermore, the levels of TG, TC, LDL, FFA, and different indexes composed of LDL/HDL, TC/HDL, TG/HDL, and TG/Glc elevated significantly in MS compared with normal rats. Thiamine improved glucose and lipid metabolism, insulin function and sensitivity, decreased the alteration percentage of body weight in MS rather than the untreated one. The level of FFA in the normal treated group was lower than in the untreated one *(P<*0*.*001*). *

**Figure 1:**
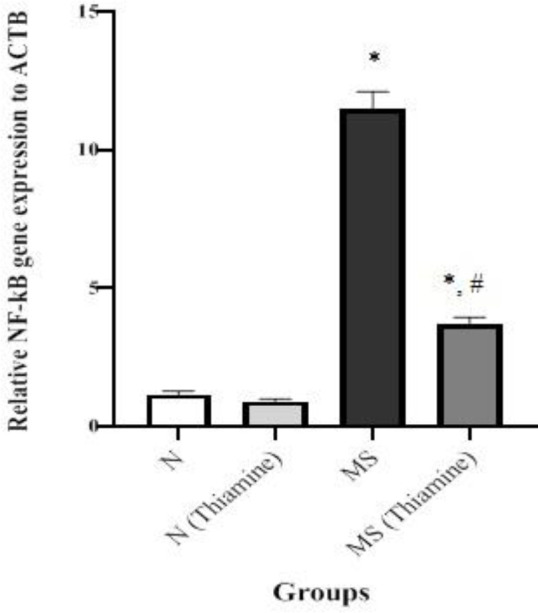
Comparison of relative hepatic nuclear factor-kβ (NF-kβ) to β-actin (ACTB) in untreated and thiamine treated normal (N) and metabolic syndrome (MS) groups

The levels of oxidative stress (AOPP, MDA, LDL oxidation products, GSH, the activities CAT and PON-1), inflammatory markers (IL-1β, MPO activity, and gene expression Nf-kβ), and glycation (g-Alb, g-LDL, MGO, and AGEs) markers as well as the activity of GLO-I in all groups, are shown in Table 2 (except for the gene expression shown in Figure 1). Levels of all cited parameters except the activities of CAT, PON-1, GLO-I increased in the MS group. Treatment corrected the activities of the cited enzymes as well as reducing glycation, oxidative stress, and inflammation markers. The levels of GSH, AOPP, MDA, LDL oxidation products, IL-1β, MGO in the normal treated group was lower than in the untreated one (*P<*0.001). 

**Figure 2:**
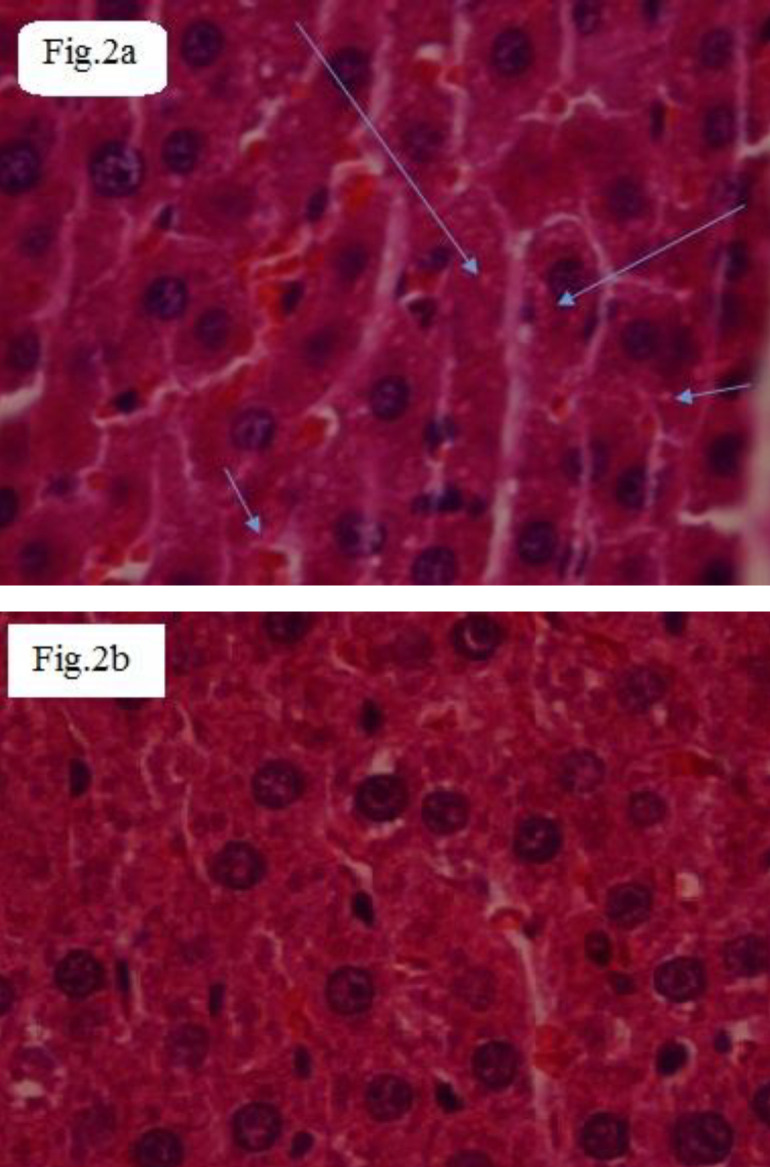
Histopathologic views (stained by H&E and original magnification ×400) of the liver in the metabolic syndrome (MS) and MS (thiamine)

 Induction of metabolic syndrome in rats increased liver weight, motivated hepatic intracellular inflammation (Figure 2), and increased activities of ALT, AST, and LDH (Table 2) in sera. Thiamine prevented hepatitis, which was confirmed by lower levels of enzymes and gene hepatic Nf-kB expression compared with the untreated group (*P<*0.001). 

In the histopathological view (Figure 2), severe intracellular inflammation, hydropic degeneration, and fatty changes have only been observed in the livers of MS rats (Figure 2a). Thiamine prevented the cited alterations in the livers of rats (Figure 2b).

**Table 1:** Effect of thiamine on FBS, insulin, HOMA-IR, and lipid profile in normal (N) and metabolic syndrome (MS) rats

Parameter	Groups (ten rats in each group)			
	N	N (Thiamine)	MS	MS (Thiamine)
Percentage of body weight alteration (%)	58.65± 3.63	59.45 ± 3.80	153.60± 10.56^*^	80.79± 5.07 ^*,^^#^
Liver weight (g)	8.70 ± 0.53	8.62 ± 0.47	9.50 ± 0.69	11.84 ± 0.73^ *,^^#^
Fasting blood sugar (mmol/L)	5.00 ± 0.25	4.92 ± 0.21	9.30 ± 0.53^*^	6.13 ± 0.45 ^*, #^
Insulin (µU/mL)	16.01± 0.83	15.93± 0.76	25.66 ± 1.64^ *^	18.50± 1.27^ *,^^#^
HOMA1	3.55± 0.19	3.84 ± 0.22	10.60 ± 0.73^*^	5.04 ± 0.41^*,^^#^
HOMA2	2.04± 0.06	2.03 ± 0.05	3.70 ± 0.12^ *^	2.47 ± 0.09^*,^^#^
%β	156.23 ± 9.80	157.77± 10.14	70.00 ± 3.42^ *^	116.57 ± 6.09^ *,^^#^
%S	48.86 ± 2.37	49.13 ± 3.39	27.00 ± 1.16^ *^	40.40 ± 1.35^ *,^^#^
Triglyceride (mmol/L)	1.37 ± 0.09	1.39 ± 0.08	2.93 ± 0.17^ *^	2.06 ± 0.11^ *,^^#^
Total cholesterol (mmol/L)	1.83 ± 0.13	1.76 ± 0.10^ *, #^	3.22 ± 0.19^ *^	2.37 ± 0.15^ *,^^#^
HDL (mmol/L)	0.94 ± 0.06	0.88 ± 0.04	0.72 ± 0.03^ *^	1.00 ± 0.07^ *,^^#^
LDL (mmol/L)	0.26 ± 0.11	0.24 ± 0.09	1.16 ± 0.07^ * ^	0.43 ± 0.11^ *,^^#^
LDL/HDL	0.28 ± 0.01	0.27 ± 0.01^ *, #^	1.61 ± 0.09^ * ^	0.43 ± 0.02^ *,^^#^
Cho/HDL	1.94 ± 0.13	2.00 ± 0.12^ *, #^	4.47 ± 0.25^ * ^	2.32 ± 0.16^ *,^^#^
TG/HDL	1.45± 0.01	1.57 ± 0.01^ *, #^	4.06 ± 0.09^ * ^	2.06 ± 0.02^ *,^^#^
TG/FBG	1.45± 0.01	1.57 ± 0.01^ *, #^	4.06 ± 0.09^ * ^	2.06 ± 0.02^ *,^^#^
Free fatty acids (µmol/L)	596.45 ± 31.91	560.00± 27.88^ *, #^	755.00 ± 41.64^ * ^	644.66 ± 35.71^ *,^^#^

^*^ Indicates significant difference with N group (*P*<0.001)

^#^ Indicates significant difference with MS group (*P*<0.001)

HOMA: homeostasis model assessment of insulin resistance; HDL: high density lipoprotein; LDL: low density lipoprotein; TC: total cholesterol; TG: triglyceride; FBG: fasting blood sugar

* Indicates significant difference with N group (*P*<0.001)

# Indicates significant difference with MS group (*P*<0.001)

**Table 2:** Comparison between levels of glycation, oxidative stress, and inflammatory markers in the normal (N), metabolic syndrome (MS) rats, and thiamine treated ones

Parameter	Groups (ten rats in each group)			
	N	N (Thiamine)	MS	MS (Thiamine)
Glycated albumin (µmol/L)	96.82 ± 5.36	85.56 ± 4.47^*,^^#^	278.38 ± 14.94^ *^	175.03 ± 8.32^ *,^^#^
Glycated LDL (µmol/L)	37.09 ± 1.78	23.91 ± 1.63^*,^^#^	113.55 ± 6.44^ *^	60.66 ± 3.87^ *,^^#^
Methylglyoxal (µmol/L)	11.62 ± 0.53	7.84 ± 0.23 ^*,^^#^	39.62 ± 2.22^ *^	18.54 ± 0.82^ *,^^#^
Advanced glycation end products (FI, A.U)	43.98 ± 2.35	29.91 ± 1.17 ^*,^^#^	307.04 ± 17.88^ *^	80.46 ± 4.13^ *,^^#^
Glyoxalase-I (U/mL)	45.23± 2.50	47.61 ± 2.68 ^*,^^#^	20.74 ± 1.38^ *^	33.42 ± 1.83^ *,^^#^
Early oxidation products of LDL (µmol/L)	12.54± 0.56	5.68 ± 0.41 ^*,^^#^	93.06 ± 5.45^ *^	40.91 ± 2.68^ *,^^#^
End oxidation products of LDL (µmol/L)	207.86 ± 12.33	185.68 ± 10.05 ^*, #^	456.83 ± 27.30^ *^	285.37 ± 22.97^ *, #^
Advanced oxidation protein products (µmol/L)	19.40 ± 1.11	13.86 ± 0.89^ *, #^	53.51 ± 3.13^ * ^	28.42 ± 1.60^ *,^^#^
Malondialdehyde (µmol/L)	12.68 ± 0.62	8.70 ± 0.49^ *, #^	136.09 ± 8.47^ * ^	58.19 ± 2.86^ *,^^#^
Glutathione (µmol/L)	181.04± 11.50	193.56 ± 12.07^*, #^	89.20± 5.19^*^	167.06 ± 9.79 ^*,^^#^
IL-1β (pg/ml)	334.30± 21.01	289.25 ± 17.88	729.59± 49.19^*^	410.82 ± 23.74 ^*,^^#^
Glyoxalase-I (U/ml)	20.74± 1.38	23.89 ± 1.09	20.74± 1.38^*^	33.42 ± 1.96 ^*,^^#^
Paraoxonase-I (U/ml)	117.03± 6.27	129.28 ± 7.15	45.64± 4.47^*^	97.05 ± 5.36 ^*,^^#^
Myeloperoxidase (U/ml)	1.40.62± 0.07	1.19 ± 0.05	1.58± 0.08^*^	3.21 ± 0.16 ^*,^^#^
Catalase (U/ml)	114.30± 7.69	119.26 ± 7.42 ^*, #^	38.50± 2.41^ *^	79.47 ± 3.76^ *,^^#^
Alanine transaminase (U/L)	23.16± 1.17	19.26 ± 0.93	84.39± 4.47^*^	27.86± 1.50 ^*,^^#^
Aspartate transaminase (U/L)	49.20± 3.26	46.43 ± 2.37	135.33± 6.72^*^	50.63 ± 2.92 ^*,^^#^
Lactate dehydrogenase (U/L)	597.53± 35.92	586.04 ± 37.51	906.52± 53.08^*^	691.74 ± 42.25 ^*,^^#^

^*^ Indicates significant difference with N group (*P*<0.001)

^#^ Indicates significant difference with MS group (*P*<0.001)

(a) Excessive intracellular inflammation, hydropic degeneration, and fatty changes were seen in the livers of MS rats.

(b) No signs of inflammation, hydropic degeneration, or fatty changes were observed in the livers of MS (thiamine) rats

## Discussion

Thiamine improved hyperglycemia, insulin resistance, and dyslipidemia in MS rats. Also, the treatment decreased body weight and glycation, oxidative stress, and inflammatory markers along with the elevation of the activity of glyoxalase-I. Moreover, thiamine prevented histopathological changes in the liver via reduction of expression of NF-kβ. 

In this study, elevation of body weight, impaired glucose tolerance, insulin resistance, and dyslipidemia in MS rat models due to four months of high sucrose solution (HSS) consumption confirms the induction of MS. Our findings of the MS rat model are similar to those of a recent paper (19).

Pro-inflammatory cytokines such as IL-1β via oxidative stress induction (37), reduction of insulin signaling and glucose transport as well as elevation of FFA and up-regulation of hepatic NF-kβ have a central role in acute hepatitis, insulin resistance, glycemia, and dyslipidemia (5, 6, 38). Also, early to end glycation products and oxidized LDL activate the NF-kβ pathway (5, 6). There is evidence that indicates high carbohydrate diet participates in thiamine deficiency (18). Besides, thiamine deficiency causes high expression of hepatic NF-kβ and hepatic inflammation. The levels of glycation products have an inverse correlation with the activity of glyoxalase-I (39). Furthermore, low activity of Glo-I leads to high expression of hepatic NF-kβ. In this study, the levels of the activators of the NF-Kβ signaling pathway including FFAs (Table 1), g-Alb, MGO, AGEs, IL-1β, glycated and oxidized LDL (Table 2), as well as expression of Nf-kβ (Figure 1) were higher in the metabolic syndrome group than in the normal group. Thiamine decreased hepatic expression of NF-kβ by reducing the cited activators in the MS rats. In the pathological view (Figures 2a and 2b), hepatic intracellular inflammation confirmed the higher expression of NF-kβ and the higher activities of ALT, AST, and LDH in the MS group rather than other groups. Further, based on liver histology, thiamine had a preventing effect against hepatic inflammation via reduction of hepatic expression of NF-kβ. The lower activities of ALT, AST, and LDH confirmed the hepatoprotective effect of the treatment. Also, the lowered level of IL-1β (Table 2) and the activity of MPO (inflammatory markers) in treated MS rats verified the anti-inflammatory property of thiamine in MS rats. The effect of thiamine on hepatic expression of NF-kβ and different activators of it in the MS group has not been reviewed yet. Lately, the ameliorative effect of thiamine on inflammation of the ruminal epithelium of Saanen goats suffering from subacute ruminal acidosis via decreased relative protein expression of IL-1β, NF-kB unit p65, and phosphorylated NF-κB unit p65, has been presented (40)**.**

MS induces glycemia, hyperinsulinemia, and insulin resistance following interfering with insulin secretion and insulin sensitivity (19). Thiamine has a cardinal role in glucose metabolism, insulin synthesis, and insulin secretion (41). Thiamine deficiency is common in MS (16, 42, 43); excess carbohydrate intake (18) as HSS, and diabetes (44) participate in enhancement of different glycation products following reduction of the activity of glyoxalase-I. Glycation products interfere in insulin secretion and function via induction of oxidative stress and inflammation (10, 45-47). In this study, thiamine decreased the levels of FBG and sera insulin along with reduction of insulin resistance by elevation of insulin function and sensitivity following the decrement of levels of IL-1β, FFA, glycation products, oxidative stress markers, and hepatic expression of NF-kβ. Previously, the lowering effect of thiamine with 2 g/L dose in drinking water on glucose and insulin resistance in Otsuka Long-Evans Tokushima Fatty (OLETF) rats has been reported (18). However, in the present study, thiamine at a much lower dose (0.18 g/L) was able to beneficially affect glucose metabolism and insulin sensitivity. Up to now, the alteration of the activity of Glo-I in MS subjects and animal models has not been presented. We reported the lower activity of Glo-I in MS rats compared with normal rats and the beneficial effect of thiamine on the activity of Glo-I. 

HSS caused obesity, dyslipidemia, and increased proneness to cardiovascular disease by elevation of different cardiovascular indices (LDL/HDL, TC/HDL, TG/HDL, and TG/FBG) in rats (Table 1). Furthermore, HSS increased lipid accumulation in the liver, resulting in liver weight gain. The liver is the main organ playing an important role in lipoprotein biosynthesis and distribution (48). Thiamine has a vital role in lipid metabolism, and its deficiency leads to dyslipidemia (49) via up-regulation of hexosamine (50) and NF-kβ pathways (6). The treatment decreased body weight and improved lipid metabolism and decreased cardiovascular indices in MS rats with down-regulation of hexosamine and NF-kβ pathways. Our study presented the beneficial effect of thiamine on diverse cardiovascular indices in the MS rat model for the first time. Thiamine probably has a regulatory role in lipid metabolism. The reducing effect of thiamine on body weight, TG, and TC in OLETE rats has been reported (18). However, in the present study, thiamine at a much lower dose was able to decrease the cited parameters. IL-1β plays a major role in fat metabolism by regulating insulin level and lipase activity (51). Therefore, reduction in its level leads to reduced insulin resistance and improved lipid metabolism (52). 

Oxidative stress is one of the major risk factors for vascular disease in MS (1). HSS induced oxidative stress in MS rats with increased MDA and AOPP along with decreased GSH and activities of CAT and PON-1 (Table 2). The treatment compensated for the cited changes and showed the antioxidant property and GSH synthesis. Recently, the benefits of thiamine on PON activity in diabetic rats has been reported (53). In the present study for the first time, the advantageous effect of thiamine on the activities of PON-1 and CAT as well as GSH level in MS rats are represented.

Glycemia, hypercholesterolemia, and oxidative stress elevate LDL glycation and oxidation, which contribute to macrovascular complications (11, 54). Our results indicated the lowering effect of thiamine on the early and end oxidation products of LDL in the normal and MS rats (Table 2). The diminishing effect of the treatment on LDL glycation and oxidation are due to its antioxidant properties as well as inducing the activities of Glo-I and PON-1. The effect of thiamine on glycation and oxidation products of LDL and activities of the cited enzymes in the MS group has not been reported up to now. 

Glycemia, oxidative stress, glycation, and thiamine deficiency are risk factors for hypertension (55). Moreover, thiamin deficiency contributes to vascular dysfunction and hypertension respectively via reduction of nitric oxide production and up-regulation of mRNAs implicated in the renin-angiotensin system. Failure to determine blood pressure in rats was a limitation of our study. However, thiamine by reducing the cited factors of hypertension could correct hypertension in MS rats. The advantageous effect of thiamine on hypertension in pre-diabetic subjects has been reported (17).

## Conclusion

Thiamine decreased body weight and improved glycemia, insulin function, dyslipidemia, and activity of glyoxalase-I in MS rats with anti-glycation, antioxidant, and anti-inflammatory activities. Furthermore, the treatment had a hepato-protective effect via reduction of NF-kβ signaling.

## Financial Support

Ardabil Medical Sciences University, Ardabil, Iran.

## Conflicts of Interest

There are no conflicts of interest
